# Selenium, Vitamin C and N-Acetylcysteine do not Reduce the Risk of Acute Kidney Injury after Off-Pump CABG: a Randomized Clinical Trial

**DOI:** 10.21470/1678-9741-2017-0071

**Published:** 2018

**Authors:** Shahram Amini, Hojat Naghavi Robabi, Mohammad Abbasi Tashnizi, Vida Vakili

**Affiliations:** 1 Department of Anesthesia, Faculty of Medicine, Mashhad University of Medical Sciences, Mashhad, Iran.; 2 Department of Anesthesiology and Critical Care, Mashhad University of Medical Sciences, Mashhad, Iran.; 3 Department of Cardiac Surgery, Mashhad University of Medical Sciences, Mashhad, Iran.; 4 Department of Community Medicine, Mashhad University of Medical Sciences, Mashhad, Iran.

**Keywords:** Acetylcysteine, Selenium, Ascorbic Acid, Acute Kidney Injury, Coronary Artery Bypass, Off-pump

## Abstract

**Objective:**

The aim of this study was to investigate the impact of perioperative administration of N-acetylcysteine, selenium and vitamin C on the incidence and outcomes of acute kidney injury after off-pump coronary bypass graft surgery.

**Methods:**

291 patients requiring elective off-pump coronary bypass graft surgery were randomized to receive either N-acetylcysteine, vitamin C and selenium 600 mg, 1500 mg, 0.5 mg, and nothing orally twice a day, respectively, from the day before to 2 days after surgery. They were assessed for the development of acute kidney injury using Acute Kidney Injury Network criteria, time of onset, its severity and duration, duration of mechanical ventilation, intensive care unit and hospital length of stay, and in-hospital mortality.

**Results:**

272 patients completed the study. The total incidence of acute kidney injury was 22.1% (n=60) with 14 (20.9%), 15 (22.1%), 21 (31.8%), and 10 (14.1%) patients in the vitamin C, NAC, selenium, and control groups, respectively (*P*=0.096). We did not register significant differences in the incidence, the time of occurrence, the severity and the duration of acute kidney injury, as well as the duration of mechanical ventilation, the intensive care unit and hospital length of stay, and the in-hospital mortality among the four groups.

**Conclusion:**

We found that perioperative administration of N-acetylcysteine, vitamin C and selenium were not effective in preventing acute kidney injury and associated morbidity and mortality after off-pump coronary bypass graft surgery.

**Table:** 

Abbreviations, acronyms & symbols				
AKI	= Acute kidney injury		ICU	= Intensive care unit
AKIN	= Acute Kidney Injury Network		K	= Potassium
BS	= Blood sugar		LOS	= Length of stay
Ca	= Calcium		MAP	= Mean arterial pressure
CABG	= Coronary artery bypass graft surgery		Mg	= Magnesium
CBC	= Complete blood count		Na	= Sodium
CHF	= Congestive heart failure		NAC	= N-acetylcysteine
CIN	= Contrast-induced nephropathy		NYHA	= New York Heart Association
COPD	= Chronic obstructive pulmonary disease		PS	= Pressure support
CPB	= Cardiopulmonary bypass		PUFA	= Polyunsaturated fatty acids
Cr	= Creatinine		RBC	= Red blood cells
eGFR	= Estimated glomerular filtration rate		RRT	= Renal replacement therapy
EuroSCORE	= European System for Cardiac Operative Risk Evaluation		SIMV	= Synchronized intermittent mandatory ventilation
Hb	= Hemoglobin level		ScVO_2_	= Central venous oxygen saturation
IABP	= Intra-aortic balloon counterpulsation			

## INTRODUCTION

Acute kidney injury (AKI) is frequently seen after cardiac surgery with dramatically increased mortality and morbidity. Cardiac surgery is the second most common cause of AKI in intensive care units (ICU)^[1]^. The incidence has been reported 0.3% to 29.7%, depending on the definition and the population under study^[2,3]^, with 1.2-3% requiring renal replacement therapy (RRT) that further increases mortality^[4-6]^.

A number of measures have been proposed to prevent the development of AKI, including maintenance of intravascular volume and adequate perfusion, and avoidance of nephrotoxic drugs^[7]^.

Several drugs, including calcium channel blockers^[8-10]^, and statins^[11,12]^ have been used to prevent AKI after cardiac surgery, but with controversial results.

Prevention of oxidative stress by antioxidants can help prevent kidney damage^[13]^. The beneficial role of antioxidants in prevention of AKI in different scenarios has been demonstrated in animal models^[14-17]^ and human models^[18]^. However, its effectiveness in preventing AKI following cardiac surgery has not been established^[19-21]^.

The purpose of this clinical trial was to investigate the role of N-acetylcysteine (NAC), vitamin C, and selenium in prevention of AKI following off-pump coronary artery bypass graft surgery (CABG). The primary outcome was to explore the incidence of AKI. Secondary outcomes included comparing AKI severity, time of occurrence and duration of AKI, need for RRT, duration of mechanical ventilation, ICU and hospital length of stay (LOS), and related in-hospital mortality among the four groups.

## METHODS

After obtaining approval from the university's Committee of Ethics and written informed consent, 291 adult patients with New York Heart Association (NYHA) class of I-III undergoing elective off-pump CABG at a teaching hospital were recruited for this clinical trial. Exclusion criteria were change from off-pump to on-pump surgery, known drug allergy, history of chronic obstructive pulmonary disease (COPD), anemia, congestive heart failure (CHF), active sepsis, preoperative ejection fraction lower than 40%, preoperative creatinine above 1.3 mg/dL, use of any nephrotoxic drugs within the last week, coronary angiography within the last 2 days, intraoperative transfusion of more than 2 units of red blood cells (RBC), perioperative use of intra-aortic balloon counterpulsation (IABP), perioperative requirement for high-dose vasopressors (defined as the use of two or more drugs to maintain adequate mean arterial pressure and perfusion, *i.e.* epinephrine or norepinephrine above 0.5 µg/kg/min, dobutamine or dopamine more than 20 µg/kg/min), and any intraoperative life-threatening events such as fatal arrhythmias, excessive bleeding, or desaturation.

Using a computer-based randomization method, patients received either selenium (n=72), vitamin C (n=73), NAC (n=73), or nothing (n=73). Patients in the selenium, vitamin C, and NAC groups received 0.5 mg, 1500 mg, and 600 mg tablets, respectively, twice a day, from 24 hours before the operation until two postoperative days.

Patients underwent standard intravenous anesthesia and were transferred to ICU for post-cardiac surgery for further monitoring and recovery, where they were under a standard monitoring and meticulous observation of a single intensive and critical care residents and fellows per shift to maintain adequate cerebral, cardiovascular, pulmonary, and renal function. A protocolized goal-directed fluid therapy with normal saline solution was conducted to achieve a mean arterial pressure (MAP) greater than 70 mmHg, lactate less than 2 mmol/L, central venous oxygen saturation (ScVO_2_) greater than 70%, and urine output greater than 1 mL/kg/hour. RBCs were transfused if hemoglobin level (Hb) was less than 8 g/dL, or Hb less than 10 mmHg in case of lactate > 4 mmol/L, ScVO_2_ < 70%, or requiring vasopressors or inotropes to keep MAP > 70 mmHg.

A protocolized intravenous paracetamol and fentanyl were used to control postoperative pain. Patients were under mechanical ventilation after entering ICU with synchronized intermittent mandatory ventilation (SIMV) and pressure support (PS) mode and weaned and extubated according to a standard weaning protocol.

Complete blood count (CBC), blood sugar (BS), sodium (Na), potassium (K), magnesium (Mg), urea, creatinine (Cr), albumin, calcium (Ca) and phosphorus were measured preoperatively. Arterial blood gases, BS, Na, K, Ca, lactate, ScVO_2_, and CBC were measured postoperatively in the first 24 hours as needed. BS, urea, Cr, Na, K and CBC were measured daily after the operation. Urine output was measured per hour during ICU stay.

AKI was diagnosed and scored based on Acute Kidney Injury Network (AKIN), defined as a serum Cr increase of 0.3 mg/dL or ≥ 1.5 times baseline within 48 hours (Table 1) (www.akinet.org).

**Table 1:** AKI staging based on AKIN criteria.

Stage	Serum creatinine criteria	Urine output criteria
Stage 1	Increase in SCr by ≥ 0.3 mg/dL or ≥ 1.5≤ 2.0 times baseline	Less than 0.5 mL/kg per hour for more than 6 hours
Stage 2	Increase in SCr by ≥ 2.0≤ 3.0 times baseline	Less than 0.5 mL/kg per hour for more than 12 hours
Stage 3	Increase in SCr by ≥ 3.0 times baseline or SCr > 4 mg/dL	Less than 0.3 mL/kg per hour for 24 hours or anuria for 12 hours

AKI=Acute kidney injury; AKIN=Acute Kidney Injury Network; SCr=serum creatinine

The estimated glomerular filtration rate (eGFR) was calculated with the Cockroft-Gault equation assuming kidney function in steady-state^[22]^. Duration (recovery) of AKI was defined as return of creatinine or glomerular filtration rate to baseline. Patients underwent RRT in case of refractory acid-base and electrolyte disorders, signs of hypervolemia or loss of consciousness attributable to hyperuremia.

Patients were discharged from ICU if they were conscious, hemodynamically stable with acceptable oxygenation and ventilation (PaO_2_/FiO_2_ > 200, PaCO_2_=35-50 mmHg), no life-threatening arrhythmias, no active bleeding, electrolyte disorders, delirium, severe anemia (Hb < 8 g/dL), and after removal of the pericardial drain.

The study was registered at Iran Registry of Clinical Trials under number IRCT2015021621098N1.

### Statistical Analysis

The sample size was calculated based on the study of Adabag et al.^[23]^ as 64 cases in each group. The sample size was increased to 292 to compensate a missing of 10%-15%.

Statistical analysis was performed using SPSS^®^ version 16 (IBM SPSS, Chicago, IL, USA). Means and standard deviation were used for normal distribution variables and median and interquartile range for otherwise. Frequencies and percentages were used for categorical variables. The ANOVA test was used to compare variables among groups. Chi-square test or Fisher's exact test was used for categorical variables. Statistical significance was considered as *P*<0.05.

## RESULTS

**Fig. 1:**
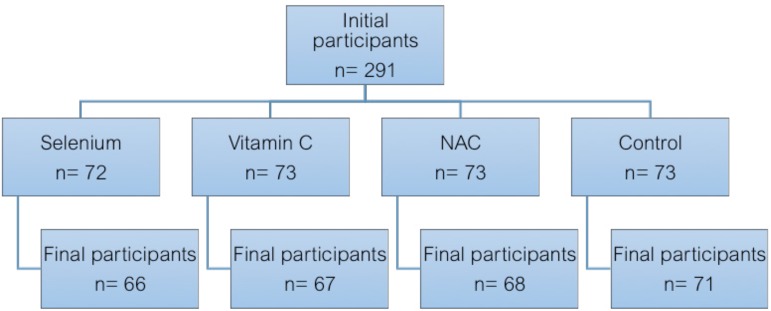
Patients' randomization, initial and final participants.

Two hundred and seventy-two patients completed the study with a mean age of 59.35±9.88 (ranging from 29 to 83) years. Of these, there were 180 males and 92 females. Two patients in the vitamin C and NAC groups were excluded for excessive bleeding and receiving more than 2 units of RBCs. One patient in the selenium group received NAC for respiratory problems. Five patients were excluded due to changing off-pump to on-pump technique intraoperatively. Eight patients received IABP intraoperatively because of hemodynamic compromise. Finally, three patients underwent on-pump CABG and received IABP simultaneously (Figure 1).

Patients were similar in relation to demographic data, comorbidities, European System for Cardiac Operative Risk Evaluation (EuroSCORE) II, cardiovascular status, baseline creatinine and eGFR (Table 2). There were no significant differences among the patients regarding the duration of surgery (4.05±0.79, 4.35±0.84, 4.17±0.82, 4.21±0.95 hours; *P*=0.225), number of grafts (3.29±0.51, 3.19±0.71, 3.25±0.76, and 3.08±0.69; *P*=0.314), units of RBC transfusion (0.68±0.66, 0.84±0.58, 0.78±0.71, and 0.58±0.71; *P*=0.114) and administered colloids (161.54±307.55, 224.26±296.55, 208.96±277.28, and 169.01±315.59; *P*=0.555) in the selenium, NAC, vitamin C, and control groups, respectively.

**Table 2:** Demographic data and clinical characteristics.

	Selenium	NAC	Vitamin C	Control	*P* value
Age (years)	58.21±10.54	60.03±10.37	60.46±10.03	58.72±8.57	0.508
Sex (male:female)	52:14:00	41:27:00	38:29:00	49:22:00	0.034
Weight (kg)	74.93±14.01	75.06±18.95	71.28±13.56	72.56±12.48	0.378
Height (cm)	166.77±9.03	164.07±10.27	164.48±9.48	165.4±10.12	0.418
Body mass index	26.92±4.62	27.82±5.91	26.14±3.45	26.54±4.31	0.190
Myocardial infarction	6 (9.09)	2 (2.94)	4 (5.97)	3 (4.22)	0.439
Hypertension	24 (36.36)	30 (44.11)	27 (40.29)	27 (38.02)	0.885
Diabetes	26 (39.39)	22 (32.35)	31 (46.26)	20 (28.16)	0.228
Hyperlipidemia	19 (28.78)	19 (27.94)	17 (25.37)	24 (33.80)	0.464
Ejection fraction	53.17±7.06	50.09±8.09	51.7±7.44	50.58±6.42	0.710
NYHA (I or II/III)	39/27	48/20	37/30	48/25	0.403
Left main/3 vessel disease	10 (15.2)	10 (14.7)	7 (10.4)	10 (14.1)	0.853
Smoking	10 (15.15)	11 (16.17)	5 (7.46)	8 (11.27)	0.416
Drug abuse	18 (27.27)	16 (23.52)	11 (16.41)	18 (25.35)	0.342
EuroSCORE	1.34±0.765	1.50±0.786	1.56±0.970	1.29±0.620	0.167
Baseline Cr	0.983±0.18	0.95±0.19	0.96±0.16	0.98±0.18	0.757
Baseline GFR	86.32±25.28	87.46±41.31	79.26±24.21	87.02±21.29	0.330
Date of angiography	25.22±29.04	28.25±46.18	31.13±43.58	24.05±21.31	0.775

Data are presented as mean ± standard deviation or numbers (percentage). Cr=creatinine; GFR=glomerular filtration rate; NAC=N-acetylcysteine; NYHA=New York Heart Association

Based on AKIN criteria, AKI was seen in 60 (22.1%) patients with no significant difference in incidence, severity, duration, and day of occurrence of AKI among the four groups (Table 3).

**Table 3:** Frequency and characteristics of AKI based on AKIN criteria.

	Selenium	NAC	Vitamin C	Control	*P* value
Frequency of AKI	21 (31.8)	15 (22.1)	14 (20.9)	10 (14.1)	0.096
Stage I	19 (31.7)	13 (21.7)	13 (21.7)	6 (10)	0.020
Stage	1 (1.7)	2 (3.3)	1 (1.7)	3 (5)	0.700
Stage III	1 (1.7)	__	__	1 (1.7)	0.570
Day of occurrence	3.05±1.62	2.80±1.37	2.57±1.60	3.40±1.64	0.677
Day of maximum severity	3.24±1.57	3.20±1.52	2.57±1.60	3.60±1.57	0.558
Frequency of recovery	16 (26.7)	9 (15)	13 (21.7)	7 (11.7)	0.228
Duration of AKI	1.86±0.81	1.78±0.97	1.23±0.43	4.29±8.26	0.465

Data are presented as mean ± standard deviation or numbers (percentage). AKI=Acute kidney injury; AKIN=Acute Kidney Injury Network; NAC=N-acetylcysteine

The mean overall duration of mechanical ventilation was 12.53±81.62 hours, with median of 5 hours. Ventilation times were 7.33±6.02, 10.68±27.15, 5.90±3.13, and 25.36±157.5 hours in the vitamin C, NAC, selenium and control groups, respectively (*P*=0.429). The mean overall ICU LOS was 2.66±3.87 days with a median of 2 days. The ICU LOS in the vitamin C, NAC, selenium and control groups was 2.36±0.86, 2.57±1.50, 2.30±0.78, and 3.20±7.36 days, respectively (*P*=0.207).

The overall mean hospital LOS was 6.45±4.17 days with a median of 6 days. Mean hospital LOS were 6.06±1.32, 6.57±2.70, 6.11±1.43, and 7.01±7.50 days in the vitamin C, NAC, selenium and control groups, respectively (*P*=0.970).

In-hospital mortality occurred in 4 (1.47%) patients, including 2 (3%), 1 (1.5%), 1 (1.5%), and zero cases in the vitamin C, NAC, selenium and control groups, respectively (*P*=0.548).

## DISCUSSION

Our study revealed that perioperative use of vitamin C, NAC, or selenium did not affect the occurrence of AKI and associated mortality and morbidity after off-pump CABG.

The protective effect of selenium for AKI has been proposed for its antioxidant effects^[19]^. Shanu et al.^[20]^ proposed that selenium could have therapeutic effects in AKI after rhabdomyolysis in the animal model. However, their findings were inconclusive. We did not find any protective effects in our study. As far as we know, there is no studies exploring the efficacy of selenium in prevention of AKI following cardiac surgery in humans.

Although we used NAC in off-pump low-risk patients, our results are consistent with other reports that suggested NAC was not effective in reducing AKI in high-risk patients undergoing on-pump^[23-25]^ and off- pump CABG^[26]^. In contrast, a meta-analysis investigating the impact of NAC, vitamin C and polyunsaturated fatty acids (PUFA) on prevention of AKI after cardiac surgery revealed that only NAC was effective^[27]^. Furthermore, in a recent study, Savluk et al.^[28]^ reported that the prophylactic use of intravenous NAC had a protective effect on renal function in patients undergoing CABG using cardiopulmonary bypass (CPB) with pre-existing moderate renal failure. Use of CPB and pre-existing renal insufficiency might explain this difference and NAC may be effective in especial group of patients undergoing cardiac surgery.

The administration of vitamin C in the prevention of contrast-induced nephropathy (CIN) after coronary angiography has been associated with different results^[29,30]^. We could not show any benefit of vitamin C in reducing the incidence of AKI. Nephrotoxicity in CIN *versus* hypoperfusion in CABG as proposed mechanisms for the development of AKI can explain the difference.

None of our patients required RRT during hospitalization. This can be attributed to the inclusion of low-risk patients, low-risk surgery, and low severity of AKI in our patients.

Our study had a few limitations as well. First, we used oral forms of the drugs with different bioavailability from parenteral forms because of high cost and resource limitations in our institution. Second, our patients were at low risk for AKI and underwent CABG without CPB. Therefore, our results cannot be extrapolated to high-risk patients or those undergoing on-pump CABG. Third, we did not use urine output for diagnosis of AKI that might lose some cases according to the existing definitions. Forth, we did not measure hemoglobin A1C to differentiate poor from well-controlled diabetes in both diabetics and non-diabetics that can affect the development of AKI as reported by Kocogulları et al.^[31]^, suggesting an association between preoperative level of Hb A_1C_ and development of AKI in non-diabetics.

In conclusion, we found that perioperative administration of NAC, vitamin C or selenium could not reduce the incidence of AKI and its associated mortality and morbidity in patients undergoing off-pump CABG.

**Table:** 

Authors' roles & responsibilities	
SA	Conception and study design; analysis and/or data interpretation; manuscript redaction or critical review of its content; final manuscript approval
HNR	Conception and study design; analysis and/or data interpretation; manuscript redaction or critical review of its content; final manuscript approval
MAT	Analysis and/or data interpretation; manuscript redaction or critical review of its content; final manuscript approval
VV	Statistical analysis; manuscript redaction or critical review of its content; final manuscript approval

## References

[1] Mao H, Katz N, Ariyanon W, Blanca-Martos L, Adýbelli Z, Giuliani A (2013). Cardiac surgery-associated acute kidney injury. Cardiorenal Med.

[2] Hoste EA, Cruz DN, Davenport A, Mehta R, Piccinni P, Tetta C (2008). The epidemiology of cardiac surgery-associated acute kidney
injury. Int J Artif Organs.

[3] Lassnigg A, Schmidlin D, Mouhieddine M, Bachmann LM, Druml W, Bauer P (2004). Minimal changes of serum creatinine predict prognosis in patients
after cardiothoracic surgery: a prospective cohort study. J Am Soc Nephrol.

[4] Mehta RH, Grab JD, O'Brien SM, Bridges CR, Gammie JS, Haan CK, Society of Thoracic Surgeons National Cardiac Surgery Database
Investigators (2006). Bedside tool for predicting the risk of postoperative dialysis in
patients undergoing cardiac surgery. Circulation.

[5] Thakar CV, Arrigain S, Worley S, Yared JP, Paganini EP (2005). A clinical score to predict acute renal failure after cardiac
surgery. J Am Soc Nephrol.

[6] Wijeysundera DN, Karkouti K, Dupuis JY, Rao V, Chan CT, Granton JT (2007). Derivation and validation of a simplified predictive index for
renal replacement therapy after cardiac surgery. JAMA.

[7] Lameire N, van Biesen W, Hoste E, Vanholder R (2009). The prevention of acute kidney injury an in-depth narrative
review Part 2: Drugs in the prevention of acute kidney
injury. NDT Plus.

[8] Antonucci F, Calo L, Rizzolo M, Cantaro S, Bertolossi M, Travaglini M (1996). Nifedipine can preserve renal function in patients undergoing
aortic surgery with infrarenal crossclamping. Nephron.

[9] Bergman AS, Odar-Cederlöf I, Westman L (1995). Renal and hemodynamic effects of diltiazem after elective major
vascular surgery: a potential renoprotective agent?. Renal Fail.

[10] Colson P, Ribstein J, Séguin JR, Marty-Ane C, Roquefeuil B (1992). Mechanisms of renal hemodynamic impairment during infrarenal
aortic cross-clamping. Anesth Analg.

[11] Billings FT 4th, Pretorius M, Siew ED, You C, Brown NJ (2010). Early postoperative statin therapy is associated with a lower
incidence of acute kidney injury after cardiac surgery. J Cardiothoracic Vasc Anesth.

[12] Liakopoulos OJ, Choi YH, Kuhn EW, Wittwer T, Borys M, Madershahian N (2009). Statins for prevention of atrial fibrillation after cardiac
surgery: a systematic literature review. J Thorac Cardiovasc Surg.

[13] Ratliff BB, Abdulmahdi W, Pawar R, Wolin MS (2016). Oxidant mechanisms in renal injury and disease. Antioxid Redox Signal.

[14] Karwasra R, Kalra P, Gupta YK, Saini D, Kumar A, Singh S (2016). Antioxidant and anti-inflammatory potential of pomegranate rind
extract to ameliorate cisplatin-induced acute kidney injury. Food Funct.

[15] Konda VG, Eerike M, Raghuraman LP, Rajamanickam MK (2016). Antioxidant and nephroprotective activities of aconitum
heterophyllum root in glycerol induced acute renal failure in
rats. J Clin Diagn Res.

[16] Long C, Yang J, Yang H, Li X, Wang G (2016). Attenuation of renal ischemia/reperfusion injury by oleanolic
acid preconditioning via its antioxidant, anti-inflammatory, and
anti-apoptotic activities. Mol Med Rep.

[17] Mahmoudzadeh L, Najafi H, Ashtiyani SC, Yarijani ZM (2017). Anti-inflammatory and protective effects of saffron extract in
ischaemia-reperfusion-induced acute kidney injury. Nephrology.

[18] Rezaei Y, Khademvatani K, Rahimi B, Khoshfetrat M, Arjmand N, Seyyed-Mohammadzad MH (2016). Short-term high-dose vitamin E to prevent contrast medium-induced
acute kidney injury in patients with chronic kidney disease undergoing
elective coronary angiography: a randomized placebo-controlled
trial. J Am Heart Assoc.

[19] Joannidis M (2007). Medical therapy of acute kidney injury. Acta Clinic Belg.

[20] Shanu A, Groebler L, Kim HB, Wood S, Weekley CM, Aitken JB (2013). Selenium inhibits renal oxidation and inflammation but not acute
kidney injury in an animal model of rhabdomyolysis. Antioxid Redox Signal.

[21] Sisillo E, Ceriani R, Bortone F, Juliano G, Salvi L, Veglia F (2008). N-acetylcysteine for prevention of acute renal failure in
patients with chronic renal insufficiency undergoing cardiac surgery: a
prospective, randomized, clinical trial. Crit Care Med.

[22] Cockroft DW, Gault MH (1976). Prediction of creatinine clearance from serum
creatinine. Nephron.

[23] Adabag AS, Ishani A, Koneswaran S, Johnson DJ, Kelly RF, Ward HB (2008). Utility of N-acetylcysteine to prevent acute kidney injury after
cardiac surgery: a randomized controlled trial. Am Heart J.

[24] Burns KE, Chu MW, Novick RJ, Fox SA, Gallo K, Martin CM (2005). Perioperative N-acetylcysteine to prevent renal dysfunction in
high-risk patients undergoing CAGB surgery: a randomized controlled
trial. JAMA.

[25] Haase M, Hasse-Fielitz A, Bagshaw SM, Reade MC, Morgera S, Seevanayagam S (2007). Phase II, randomized, controlled trial of high-dose
N-acetylcysteine in high-risk cardiac surgery patients. Crit Care Med.

[26] Song JW, Shim JK, Soh S, Jang J, Kwak YL (2015). Double-blinded, randomized controlled trial of N-acetylcysteine
for prevention of acute kidney injury in high ris patients undergoing
off-pump coronary artery bypass. Nephrology.

[27] Ali-Hassan-Sayegh S, Mirhosseini SJ, Tahernejad M, Mahdavi P, Shahidzadeh A, Karimi-Bondarabadi AA (2016). Impact of antioxidant supplementations of cardio-renal protection
in cardiac surgery: an updated and comprehensive meta-analysis and
systematic review. Cardiovasc Ther.

[28] Savluk OF, Guzelmeric F, Yavuz Y, Cevirme D, Gurcu E, Ogus H (2017). N-acetylcysteine versus dopamine to prevent acute kidney injury
after cardiac surgery in patients with preexisting moderate renal
insufficiency. Braz J Cardiovasc Surg.

[29] Spargias K, Alexopoulos E, Kyrzopoulos S, Iokovis P, Greenwood DC, Manginas A (2004). Ascorbic acid prevents contrast-mediated nephropathy in patients
with renal dysfunction undergoing coronary angiography or
intervention. Circulation.

[30] Zhou L, Chen H (2012). Prevention of contrast-induced nephropathy with ascorbic
acid. Intern Med.

[31] Kocogullari CU, Kunt AT, Aksoy R, Duzyol C, Parlar H, Saskin H (2017). Hemoglobin A1c levels predicts acute kidney injury after coronary
artery bypass surgery in non-diabetic patients. Braz J Cardiovasc Surg.

